# Extracorporeal Membrane Oxygenation for Acute Toxic Inhalations: Case Reports and Literature Review

**DOI:** 10.3389/fmed.2021.745555

**Published:** 2021-09-29

**Authors:** Dun Yu, Zhang Xiaolin, Pan Lei, Li Feng, Zhang Lin, Shen Jie

**Affiliations:** ^1^Department of Emergency and Critical Care Medicine, Jinshan Hospital, Fudan University, Shanghai, China; ^2^Department of Respirology, Shanghai Public Health Clinical Center, Fudan University, Shanghai, China; ^3^Department of Critical Care Medicine, Medical Research Center of Chemical Injury, Jinshan Hospital, Fudan University, Shanghai, China

**Keywords:** extracorporeal membrane oxygenation, acute toxic inhalation, poisoning, ARDS, cardiogenic shock, cardiac arrest

## Abstract

Previous studies have shown that poisoning is a major threat to human health. Inhalation of acute toxic gas has been linked to serious health consequences. Among the antidotes for poisoning currently used, supportive care is the most common intervention in clinical practice. Severe acute respiratory distress syndrome (ARDS) and/or refractory cardiogenic shock or cardiac arrest caused by toxins are associated with high mortality and are difficult to treat. Extracorporeal membrane oxygenation (ECMO) is an aggressive supportive measure used to manage severely poisoned patients. This study presents two cases of acute toxic gases inhalation, severe ARDS and circulatory instability induced by bromine inhalation, and ARDS induced by nitric acid inhalation which were successfully treated with ECMO. The ECMO techniques used in the animal models and in human cases to treat severe poisoning are described as well as the indications, contraindications, complications, and weaning of ECMO.

## Introduction

Poisoning causes a detrimental effect to human health. The 37th Annual Report of American Association of Poison Control Center's (AAPCC) National Poison Data System (NPDS) showed that there were 2,148,141 cases of human toxicological exposure in 2019. In addition, 2,048 out of 2,619 deaths were identified as exposure-related fatalities whereas 1,688 (81.4%) of the 2,048 fatalities were identified as drug exposure cases. The main exposure routes were ingestion (80.1%), inhalation/nasal (8.15%), and parenteral (5.24%). Furthermore, the most human toxic exposures were unintentional (76.6%) (1). The Centers for Disease Control and Prevention (CDC) reported that the incidence of mortality due to poisoning has been increasing in the past decade (2).

The previous studies define acute toxicity as the adverse effects of a substance resulting either from single or multiple exposures in short periods of time (usually less than 24 h) (3, 4). Acute toxic inhalation is considered an emergency in clinical practice. The previous studies have established that smoke, gases, and vapors are the most frequently inhaled substances (4, 5). Acute inhalation of toxic substances often occurs during the production, operation, storage, transportation, and other human factors. A National Occupational Exposure Survey (NOES) conducted from 1981 to 1983 estimated that more than 1,000,000 million workers in the United States were at risk of exposure to respiratory irritants annually. However, the data from poison control centers have suggested that exposure to the toxic substances occurs more frequently in the home environment than at workplaces (6).

Inhalation of gases, mists, aerosols, fumes, or dust may irritate the lungs, cause acute respiratory distress syndrome (ARDS), asphyxiation, and cardiogenic shock (5). Respiratory failure or cardiogenic shock or cardiac arrest caused by acute toxic inhalation are related to significant mortality (7–9). Several antidotes have been used to control the damage caused by some toxins, such as hydroxocobalamin for cyanide, fomepizole for methanol, pralidoxime chloride, and atropine for organic phosphorus pesticide poisoning, and oxygen for carbon monoxide. However, such antidotes are not effective in all the cases. Extracorporeal membrane oxygenation (ECMO) is an external device that can provide cardiopulmonary support for patients. The previous studies reported successful use of ECMO in the treatment of severe ARDS (10, 11) and refractory cardiogenic shock or cardiac arrest (12–14) following its introduction. The studies have also reported the use of ECMO in both animal models and human cases with refractory shock and/or ARDS induced by intoxication or toxicant exposure (15–19). The randomized trials of ECMO in the poisoned patients with acute toxic inhalation have not yet been undertaken. The available evidence has been generated from observational cohorts, case series, and case reports (20). The previous studies have, however, reported that early initiation of ECMO can improve the outcome of severely poisoned patients when optimal conventional treatment failed (18, 21, 22). Therefore, ECMO is a potential treatment option for patients with acute toxic inhalation with refractory circulatory shock and/or ARDS. The previous studies have shown that ECMO helps in the recovery from acute incidents, or transition to or candidacy for long-term advanced therapies, such as surgical ventricular assist devices or transplants (14, 23, 24).

In the current study, two successful ECMO support cases for acute toxic gases inhalation with severe ARDS were elaborated. In addition, the current study described the ECMO techniques, an application of ECMO in poisoned patients, indications, contraindications, complications of use, and weaning of ECMO.

## Case Reports

### Case 1

A 44-year-old man, working in a chemical plant, was accidentally exposed to bromine gas (Br_2_). The worker became unconscious for 15 min and was transferred to an open-air setting by the colleagues, where the man gained consciousness after 10 min.

**Figure 1:**
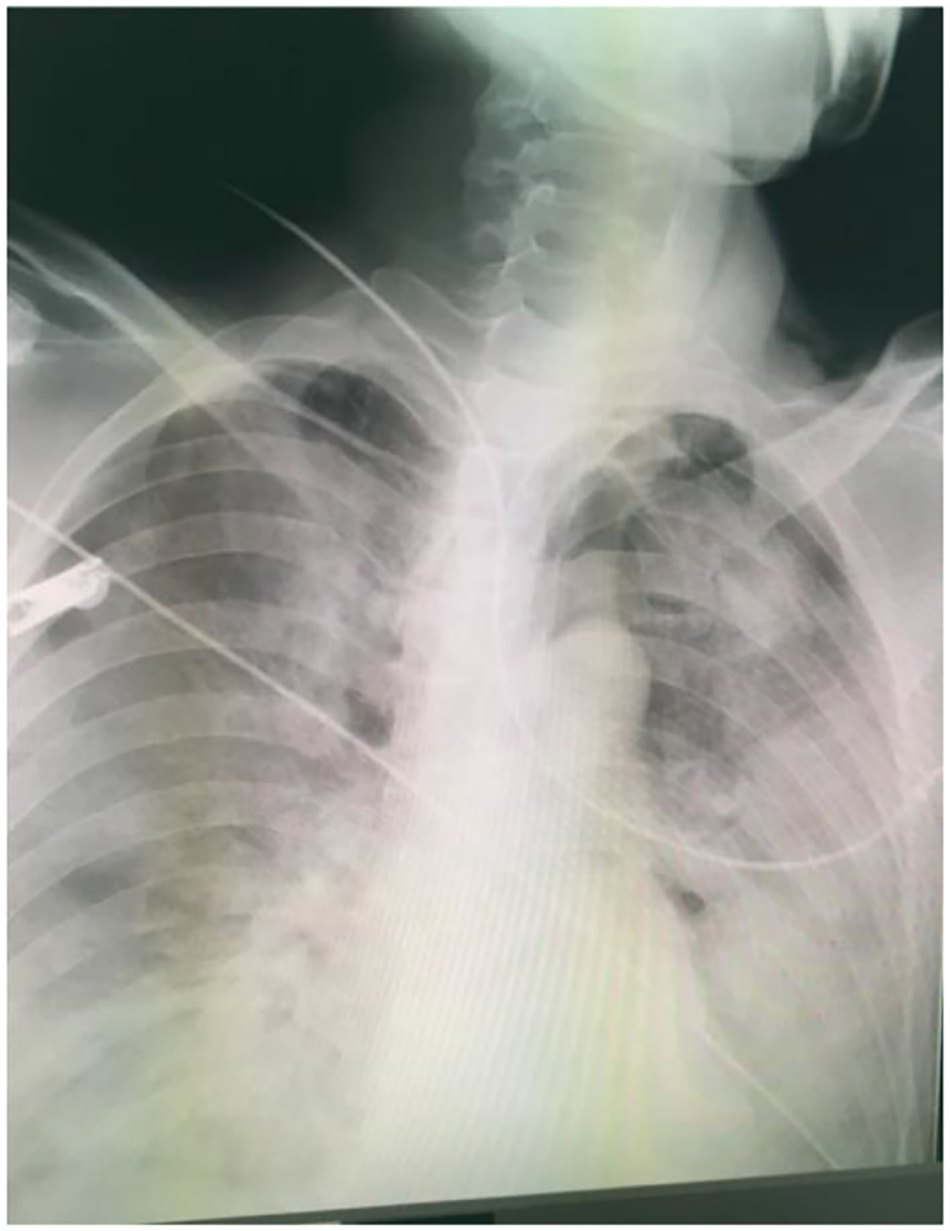
Chest x-ray revealed pulmonary edema with fluid-filled bilateral lungs.

Upon admission to a local hospital, the man presented with several symptoms, such as dyspnea, vomit, fatigue, cough, pharyngalgia, and mental confusion. The patient remained conscious with the following vital signs: blood pressure 92/63 mmHg, pulse rate 94 beats/min, respiratory rate 22 breaths/min, temperature 36°C, oxygen saturation (80–85%) supported by mask ventilator assisted ventilation with inhaled 100% oxygen concentration. Arterial blood gases obtained before intubation were: pH 7.309, partial pressure of oxygen (PaO_2_) 8.18 kpa, partial pressure of carbon dioxide (PaCO_2_) 6.73 kpa, and bicarbonate concentration −0.9 mmol/L (P/F oxygen ratio was 61.5 mmHg). Despite inhaling 100% oxygen, the status of dyspnea did not improve but progressively worsened accompanied with profuse sweating and irritability. Moreover, a pronounced stridor could be heard. The physical examination revealed cyanosis of the lips and mouth, shortness of breath, three concave signs, and increased bilateral vesicular sounds. Because of laryngeal edema caused by Br_2_ irritation and potential retention of secretions in the lower respiratory tract with a probable need for more than a week of respiratory support, a tracheotomy was performed immediately. A large amount of pinkish foamy secretions was discharged from the patient's mouth after tracheotomy. The clinical and laboratory investigations indicated that the pulse oxygen saturation (SpO_2_) was less than 90% after assisted mechanical ventilation. A chest x-ray showed pulmonary edema with fluid-filled bilateral lungs (Figure 1). Subsequently, a single dose of methylprednisolone (80 mg) was administered intravenously. Considering the severity of the Br_2_-induced injury, the patient was transferred to critical care center 4 h after Br_2_ inhalation for definitive treatment.

At the critical care center, the patient received synchronized intermittent mandatory ventilation (SIMV), with initial settings of positive end-expiratory pressure (PEEP) of 12 cmH_2_O, fraction of inspired oxygen (FiO_2_) of 1.0, respiratory rate (*RR*) of 16 bpm, tidal volume (V_T_) of 4 ml/kg, and plateau pressure (Pplat) ≤ 25 cmH_2_O. In addition to the routine critical care, initial management included absolute bed rest, intravenous methylprednisolone, anticoagulation, energy and vitamin supplements, maintenance of water, electrolytes, and acid-base balance. However, the condition of the patient worsened. Arterial blood gases recorded 48 h after mechanical ventilation were: pH 7.26, PaO_2_ 6.13 kpa, PaCO_2_ 4.97 kpa, and bicarbonate concentration 2.4 mmol/L (P/F oxygen ratio was 46 mmHg). Thereafter, the patient was initiated on ECMO. Two cannulas were placed percutaneously by vessel puncture, guidewire placement, and serial dilation. One cannula (Edward 24F, Edwards Lifesciences Corp., CA, USA) was advanced into the right femoral vein; another (Edward 16F) into the right internal jugular vein. The assembled circuit (PLS heparin-coated ECMO kit, Edward) was primed. Initial ECMO flow settings were: blood flow of 4 L/min, sweep gas flow of 2 L/min, FiO_2_ of 1.0, and temperature of the water bath was set at 36.8°C (adjusted according to arterial blood gas). The ventilator settings immediately pre-ECMO were: P-SIMV, PEEP 12 cmH_2_O, FiO_2_ 0.5, RR 8 bpm, V_T_ 4 ml/kg, Pplat ≤ 25 cmH_2_O. The patient was sedated with fentanyl and midazolam during cannulation and management for the first 12–24 h. Richmond Agitation-Sedation Scale (RASS) was −5. The neurologic examination was performed daily to ensure that the sedation was sufficient. Once the patient had stabilized on ECMO, all the sedatives and narcotics were stopped and resumed depending on the levels of anxiety and discomfort of the patient. Heparin (50–100 units per kg) was administered at the time of cannulation and continuously infused during ECMO. Heparin infusion was regulated to keep the activated partial thromboplastin time (APTT) at designated levels (usually 1.5 times the normal values for the APTT measurement system). Hemoglobin levels, blood platelet counts, and lactic acid accumulation were regularly detected to monitor the development of complications. The rest settings during ECMO support were: Ppeak 20–25 cmH_2_O, PEEP 10–15 cmH_2_O, RR 10 bpm, and FiO_2_ 0.4.

**Figure 2:**
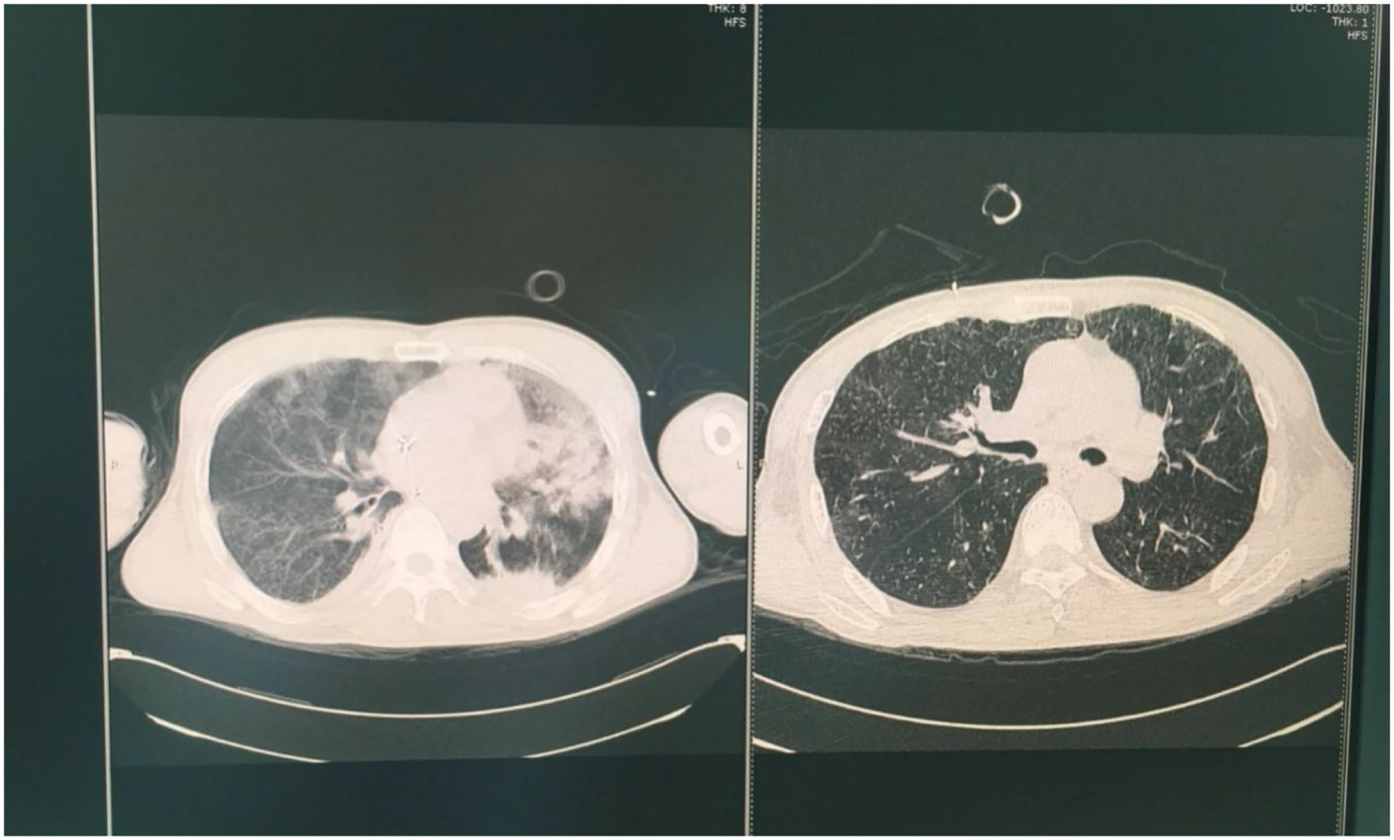
The chest CT images on the second **(left)** and seventh day **(right)** after weaning.

In addition to ECMO support, the pharmacologic diuresis and antibiotic treatment were administered. Respiratory parameters of the patient improved and the chest CT images showed that the bilateral infiltrations had regressed after 7 days of therapy. The patient was weaned off ECMO upon shock reversal and attaining stable condition. The arterial blood gases were analyzed when the patient was extubated: pH 7.47, PaO_2_ 98.3 mmHg, PaCO_2_ 38.7 mmHg, and SpO_2_ 99%. The chest CT was performed on 2nd and 7th day after weaning (Figure 2). It was found that the lung edema had resolved. Follow-up chest CT after discharge from the hospital showed progressive improvement in the affected lung regions.

### Case 2

A 41-year-old man with a history of hepatitis B, who was taking tenofovir disoproxil fumarate drugs, presented with the complaint of chest stuffiness and shortness of breath immediately after inhaling mixed chemical gas during unloading of concentrated nitric acid. The man was generally healthy with a normal hepatic function. However, the serum HBV-DNA level of this man was lower than the detection limit. Based on the reports from the factory, the mixed gas mainly comprised of NO_2_, NO, and HNO_3_, with a small amount of benzene.

**Figure 3:**
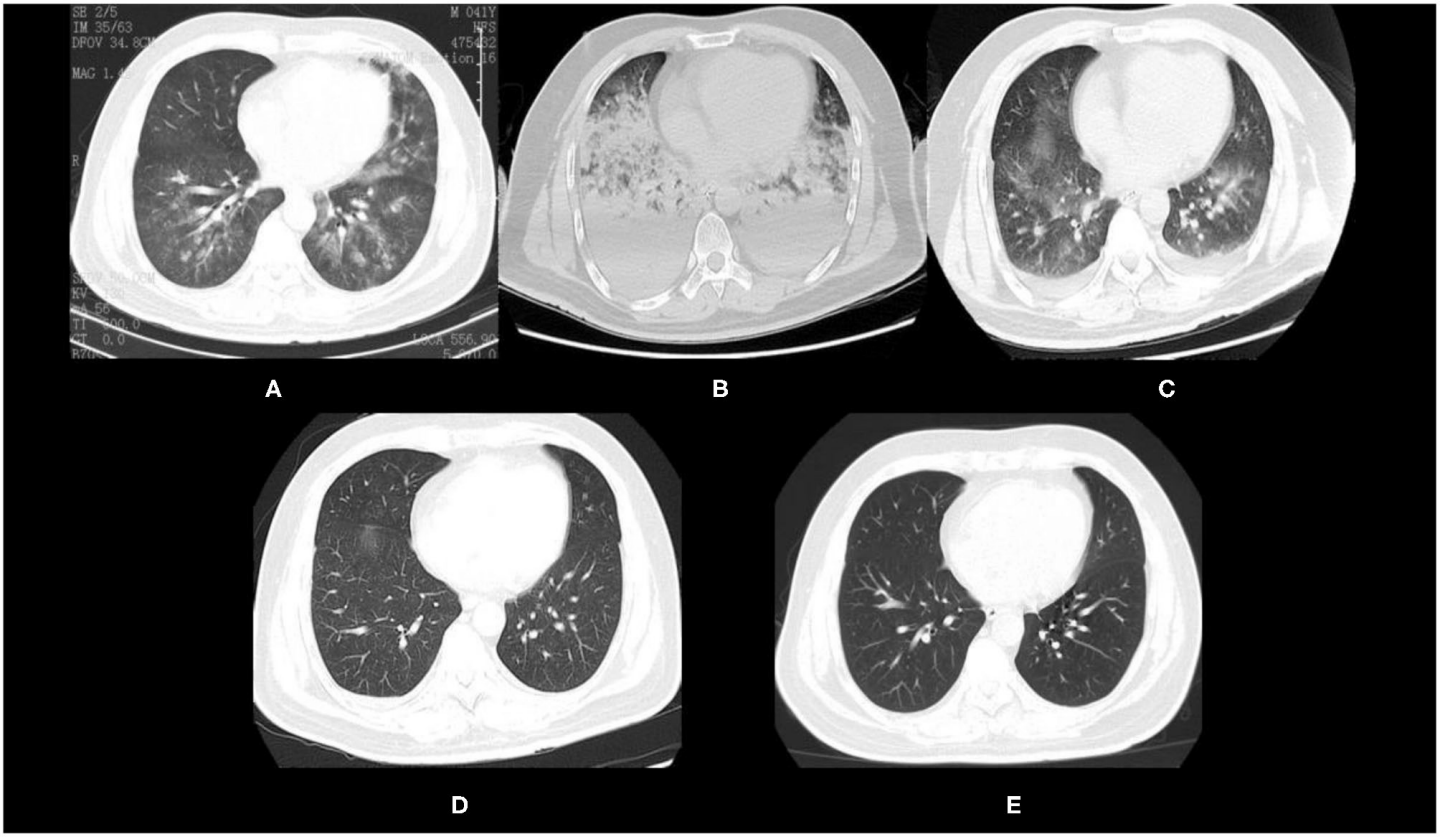
Chest CT of the patient after mixed chemical gas inhalation. **(A)** Chest CT 2 h after inhalation. **(B)** Chest CT 20 h after ECMO initiation. **(C)** Chest CT after ECMO was weaned off. **(D)** Chest CT 2 days after weaning. **(E)** Chest CT 54 days after discharge.

Upon admission to a local hospital, the oxygen saturation was 94% after receiving nasal cannula oxygenation at a flow rate of 2 L/min. The symptoms of shortness of breath worsened 2 h after the nasal cannula oxygenation. Chest CT showed scattered exudation in bilateral lower lungs (Figure 3A). The patient was transferred to a local tertiary hospital where the man received high-flow oxygenation. Non-invasive ventilation was administered 14 h following the failure of high-flow oxygenation. At 9 h after admission to the intensive care unit (ICU), the patient was orotracheally intubated and mechanically ventilated due to progressive hypoxemia. However, the partial pressure of oxygen (PaO_2_) dropped to 50 mmHg (P/F oxygen ratio was 62.5 mmHg) 4.5 h after mechanical ventilation. Chest CT showed extensive exudation and pleural effusion in bilateral lungs (Figure 3B). These findings indicated ARDS and veno-venous-ECMO (VV-ECMO) was performed immediately.

Two cannulas (23-19Fr) were advanced percutaneously *via* the right femoral vein for drainage and the right internal jugular vein for reinfusion. The assembled circuit (PLS heparin-coated ECMO kit, Maquet, Germany) was primed. The initial ECMO flow settings were: blood flow of 4.5 L/min, sweep gas flow of 4.5 L/min, FiO_2_ of 1.0, temperature of the water bath was set at 36°C (adjusted according to arterial blood gas). The ventilator settings immediately pre-ECMO were: P-SIMV, PEEP 10 cmH_2_O, FiO_2_ 0.3,RR 10 bpm, V_T_ 4 ml/kg, and Pplat ≤ 25cmH_2_O. The patient was sedated with remifentanil and midazolam during cannulation and management for the first 12–24 h. RASS was −5. The neurologic examinations were done daily to ensure that the sedation was sufficient. Heparin (50–100 units per kg) was administered at the time of cannulation, and then through continuous infusion during ECMO. Heparin infusion was regulated to keep APTT within designated levels (usually 1.5 times normal for the APTT measurement system). The hemoglobin levels, blood platelet counts, and lactic acid accumulation were measured regularly to monitor the occurrence of complications. The rest settings during ECMO support were: Ppeak 20–25 cmH_2_O, PEEP 10–15 cmH_2_O, RR 10 bpm, and FiO_2_ 0.4.

In addition to ECMO, the patient received intravenous methylprednisolone, prone ventilation, and nasogastric gavage with N-acetylcysteine and pirfenidone. The patient was weaned off successfully 71 h after ECMO support and was extubated 16 h later. Chest CT (Figure 3C) showed that the diffuse exudation and pleural effusion in bilateral lungs had significantly resolved. The chest CT revealed progressive remission of exudative lesions in bilateral lungs 2 days after weaning (Figure 3D). The patient was discharged from the hospital 4 days after weaning. At 54 days after discharge, a follow-up chest CT showed that the lesions in bilateral lungs had almost resolved (Figure 3E). Lung function of the patient was normal 20 months later. The patient currently continues running and is said to have completed a full marathon recently.

## ECMO Techniques

Extracorporeal membrane oxygenation, an auxiliary technique for respiratory and circulatory support, is increasingly being applied in clinical practice. ECMO drains the hypoxic blood from the venous system through the venous cannula. Then, the blood is oxygenated by a membrane oxygenator and pumped back to the patient through a second cannula (9, 25, 26). There are two ECMO modalities; VV-ECMO and veno-arterial-ECMO (VA-ECMO). In VV-ECMO, the blood is drawn from the peripheral vein, often femoral vein, oxygenated and decarboxylated in a dedicated extracorporeal rotor/oxygenator device and pumped back to the right atrium through a cannula. However, VV-ECMO only provides respiratory support and is primarily used in ARDS patients (9, 26). In VA-ECMO, hypoxic blood is drawn from the vicinity of the right ventricle through a large bore cannula, which is usually percutaneously placed through the right jugular or femoral veins. The femoral vein is especially useful in emergency settings, for example, when cardiopulmonary resuscitation (CPR) is performed and chest compressions prevent proper, hygienic placement of the catheter through the right jugular vein. Then, the blood is pumped through the oxygenator and returned to the aorta *via* a large arterial catheter. VA-ECMO provides both respiratory and circulatory support and can be used in hemodynamically compromised patients. Therefore, ECMO is a potentially effective treatment modality for severely poisoned patients with severe ARDS and refractory cardiogenic shock or cardiac arrest.

## ECMO for Acute Toxic Inhalation

In toxicological studies, ECMO has shown positive effects in both the animal experiments and clinical cases. In the 1990s, this technique was found to significantly improve the survival of animals undergoing cardiac arrest after drug intoxication. Freedman et al. (16) reported that all lidocaine-induced cardiac arrest dogs survived through ECMO support, while the dogs treated with standard resuscitation had a mortality rate of 75%. In recent years, ECMO has also achieved promising results for the treatment of chemical gas poisoning-induced cardiac arrest animals. Simonsen et al. (27) treated carbon monoxide (CO)-poisoned pigs with ECMO and conventional mechanical ventilation, and found that ECMO significantly reduced the incidences of cardiac arrest and mortality in CO-poisoned pigs, when compared with the conventional mechanical ventilation group. Furthermore, after sequential ECMO treatment, the survival rates of conventional mechanical ventilation group were found to have improved. In a previous study, the Danish scientists successfully cannulated and established VA-ECMO for CO-induced cardiac arrest in the porcine models during airborne transportation (28). Although the animal study findings show that ECMO is effective in toxic gas-induced cardiovascular compromise, the experiments using these models are not the same as real clinical settings. Therefore, the role of ECMO in the treatment of acute toxic inhalation should be explored further in the clinical studies.

The animal model as well as human case reports and case series have shown that ECMO has favorable outcomes for acute toxic inhalation. Smoke is a common toxin that causes acute inhalation injuries and ARDS that requires ECMO support. The cases of ECMO support for fire-induced smoke inhalation injuries (29–35) and zinc chloride inhalation from smoke bombs have been reported (36). Electronic cigarettes (e-cigarettes) are battery-powered devices that aerosolize various substances for inhalation, such as nicotine, tetrahydrocannabinol, cannabidiol, and flavoring agents that may contain diacetyl. However, e-cigarettes should be evaluated further because they also cause pulmonary toxicity (37). As of November 13, 2019, a total of 2,172 “e-cigarette or vaping product use-associated lung injury” (EVALI) cases had been reported to the CDC of the Unites States, with 42 confirmed deaths (1). Landman et al. (38) reported a case of vaping-associated severe acute bronchiolitis, which caused near-fatal hypercapnic respiratory failure requiring intubation and ECMO in a 17-year-old male. The patient was weaned from VV-ECMO and ventilator, tracheostomy tube removed, and was discharged after 47 days in hospital. Accidental powder inhalation is a potential problem for infants. Panarello et al. (39) reported a case of severe ARDS due to accidental inhalation of rice starch powder in a 17-month-old girl. The girl was successfully treated with VV-ECMO. Metal fume inhalation also causes an acute respiratory and circulatory failure, and ECMO has been successfully applied for the severely poisoned patients (40, 41). Toxic gas inhalation causes lung damage. ECMO is a salvage therapy for inhaled toxic gases, such as, ammonia (42), hydrofluoric acid (43), hydrochlogen chloride (44), volatile hydrocarbons (45), carbon monoxide (46, 47), phosgene (48), chlorine (49–51), humidifier disinfectants (52), nitric and hydrofluoric acids (53, 54), and aluminum phosphide (55).

We report two cases of successful ECMO treatment for toxic volatile chemical inhalation. Br_2_, which is a reddish-brown fuming liquid with a unique odor and volatile at room temperature, is widely used as the raw material for the synthesis of pharmaceutical compounds, flame retardants, dyes, photographic chemicals, bleaches, and disinfectants. Br_2_ causes damage to the eyes, skin, central nervous system, and respiratory system (56–58). Br_2_ and hypobromous acid (HOBr), its hydrolysis product, are strong oxidants that initially react with antioxidants in the lung epithelial lining fluid after inhalation. The depletion of antioxidant stores promotes the reaction of Br_2_ and HOBr with plasma membranes of lung epithelial cells to form reactive intermediates, such as brominated lipids, which injure the distal sites. Moreover, Br_2_ inhalation promotes the intravascular hemolysis. The ensuing elevated free heme causes acute lung injury due to increased acute oxidative stress and inflammation in the lung tissues (59–61). During ECMO, we regularly monitored the hemoglobin levels, however, we did not observe intravascular hemolysis. Maybe, the heme levels could have been elevated, but the elevated level did not attract our attention. The inflammatory responses due to Br_2_ exposure worsens the initial pulmonary and systemic injuries, which in turn, aggravates the lung damage due to released inflammatory mediators. Inhalation of Br_2_ leads to various pulmonary symptoms, such as cough, dyspnea, hypoxia, or even death due to respiratory failure in the adults (56). There is no specific antidote for Br_2_ inhalation. Therefore, the first intervention step is to quickly move the patient out of the toxic environment, followed by the administration of appropriate therapies for symptomatic and supportive care, such as assisted ventilation, bronchodilators, and antibiotics. In our case, the patient was unresponsive to the conventional treatment, which prompted the initiation of ECMO for cardiopulmonary support. After 7 days of ECMO treatment, the condition of the patient improved and then, was successfully weaned off the treatment. To the best of our knowledge, this is the first reported case of successful ECMO treatment for Br_2_ inhalation-induced ARDS.

Nitric acid is a strong acid and an oxidizing agent for various applications. One of its main uses include the production of ammonium nitrate in the fertilizer industry and other industrial applications. Pure HNO_3_ is a colorless liquid with a boiling temperature of 84.1°C and can partially decompose to form nitrogen dioxide (NO_2_). When exposed to air, pure HNO_3_ releases white fumes while HNO_3_ admixed with NO_2_ liberates reddish-brown vapors (62, 63). The applications of HNO_3_ generate various oxides of nitrogen, such as nitic oxide (NO), dinitrogen trioxide (N_2_O_3_), dinitrogen tetroxide (N_2_O_4_), and dinitrogen pentoxide (N_2_O_5_) (63). The inhalation injuries attributed to HNO_3_ and its oxidized derivatives have been shown to cause acute local tissue inflammation within the lower respiratory tract (63). With regards to the human exposure, NO_2_ is the most important nitrogen oxide. Specific mechanisms leading to lung injury following HNO_3_ exposure have not been fully elucidated. However, it has been postulated that these injuries are due to a combination of free radical injuries, NO_2_ generation of nitric acid after mucosal membrane contact, decrease in α-1-progease inhibitor, lipid peroxidation, thiol oxidation, and 3-nitrotyrosine formation (64). These deleterious effects lead to slough of tracheobronchial mucosa and are frequently accompanied by the direct toxic effects to the airways at the cellular level, which trigger the inflammatory cascade responses. The symptoms of HNO_3_ inhalation injury have been generalized into three phases, namely, acute, subacute, and delayed onset phases (63). In this study, acute exposure led to an immediate onset of chest tightness and shortness of breath. Subsequently, the patient presented with subacute symptoms, such as dyspnea and generalized weakness. Then, within 24 h after exposure, the patient quickly presented with delayed symptoms, such as dyspnea, tachypnea, bronchospasm, and cyanosis, which indicated pulmonary edema and ARDS. The symptomatic treatment of lung inhalation injury from HNO_3_ has been shown to be largely supportive, and it remains unstandardized (63). Kido et al. (65) reported a case of HNO3-induced pulmonary injury with improvement after corticosteroid administration. Meaden et al. (63) reported a case of pulmonary edema occurring after HNO_3_ inhalation, which improved after the bronchodilator treatment. We report the first case of successful ECMO treatment for ARDS after HNO_3_ inhalation, thereby, providing a new treatment modality for HNO_3_ inhalation-induced ARDS.

Of note, differences in the application of VV-ECMO for the management of toxic gas inhalation and other conditions should be noted. The patients with toxic gas inhalation are more prone to secondary infection and sepsis due to damage of the respiratory tract caused by the toxic gases compared with the patients with other ECMO indications. Therefore, monitoring body temperature, complete blood count, procalcitonin (PCT), and other infection indicators should be carried out during ECMO management. Full caloric and protein nutritional support are essential. High-dose, short-course methylprednisolone was administered in the early stages of both the cases. Although there was no evidence of reduced mortality, it improved the conditions of patients in our cases. In case 1, the patient was subjected to tracheostomy for laryngeal edema, therefore, the patient was at risk of infections. Appropriate antibiotics were administered to prevent the infections. Pharmacologic diuresis is important for edema clearance. In case 2, the patient was subjected to prone ventilation, which may have a positive effect on the rapid recovery from ARDS. The percutaneous cannulations were performed through the right femoral vein and the right internal jugular vein. Heparin was administered at the cannulation time and continuously infused during ECMO. Two patients were sedated. After the patient had stabilized on ECMO, all the sedatives and narcotics were stopped and resumed depending on the levels of anxiety and discomfort of the patient.

Although an increasing number of cases report the successful use of ECMO for acute toxic inhalations, evidence is majorly from the case reports and case series. We conclude that, when optimal conventional treatments fail, ECMO is a potential treatment modality for severe ARDS induced by acute toxic inhalations. However, large observational studies and randomized clinical trials should be conducted to support the effects of ECMO.

## Indications, Contraindications, and Complications for ECMO in Poisoned Patients

Extracorporeal membrane oxygenation provides effective gas exchange, reduces mechanical ventilation intensity, allows adequate lung rest, and improves patient outcomes. With the increasingly mature clinical applications of ECMO, there are many successful applications of ECMO in patients with irritant gas poisoning. ECMO improves the prognostic outcomes for severe hypoxemia and severe decompensated hypercapnia under optimal mechanical ventilation. Currently, this technique is a popular treatment option for medical toxicologists. However, it is not a standard treatment alternative as it lacks therapeutic evidence from large poisoning-based observational studies and randomized clinical trials. Indications for the poisoned patients are still under investigation. It has been recommended that ECMO can be initiated as soon as severely poisoned patients become unresponsive to optimal conventional interventions and have no contraindications for ECMO support.

### Indications

Veno-venous-extracorporeal membrane oxygenation is recommended for respiratory failure when cardiac function is adequate or moderately depressed in the poisoned patients. It is also indicated for when the risk of mortality is greater than or equal to 80% (66). Approximately 80% mortality is associated with PaO_2_/FiO_2_ < 100 on FiO_2_ > 90% and/or Murray score 3–4, age-adjusted oxygen index (AOI) >80, age, PaO_2_/FiO_2_ ratio, and plateau pressure (APSS) of eight despite optimal care for 6 h or less (66–69). VA-ECMO is recommended for poisoned patients with refractory cardiogenic shock or cardiac arrest and/or ARDS who are unresponsive to resuscitation, high-dose of vasopressors, transcutaneous cardiac pacing, and intra-aortic balloon pump (IABP), to provide cardiopulmonary support and maintain end-organ perfusion.

### Contraindications

Few absolute contraindications for ECMO have been reported. They include severe irreversible non-cardiac organ failure limiting survival (e.g., severe anoxic brain injury or metastatic cancer), and irreversible cardiac failure if the transplantation or long-term ventricular assist devices are not considered (70). Moreover, ECMO treatment is absolutely contraindicated in the preexisting or acute conditions that are incompatible with recoveries, such as neurologic injury or end-stage malignancy that preclude a meaningful chance of intermediate-term survival or functional recovery (14). The relative contraindications for ECMO include severe coagulopathy or contraindications for systemic anticoagulation, such as advanced liver disease. Limited vascular access (severe peripheral arterial disease, extreme obesity, and amputated limbs), central as well as axillary cannulation are considered alternatives. Unrepaired aortic dissection, in which VA-ECMO flow may cause the additional fenestrations or propagate dissection flaps, should be cautiously performed, and acute aortic insufficiency that cannot be surgically corrected almost immediately is prohibited (14). Other relative contraindications include mechanical ventilation at high settings (FiO_2_ > 90%, plateau pressure > 30 cmH_2_O) for 7 days or more and major pharmacologic immunosuppressions (absolute neutrophil count <400/mm^3^). Even though the increasing age is associated with increased risks, no specific age contraindications have been reported (69).

### Complications

Although ECMO has many clinical benefits, it also has notable complications. Severe potential complications include bleeding, thromboembolism, neurological injury, infection, limb ischemia, acute kidney injury, and homolysis (9, 14, 70–72).

## Weaning

There is no universal method for determining whether ECMO can be successfully weaned and decannulated, however, some general principles apply.

In VV-ECMO, ECMO flow is decreased in steps to 1 L/min at sweep FiO_2_ 100% or decreased to 2 L/min, then sweep FiO_2_ is decreased to maintain SaO_2_ > 95%. When SaO_2_ is stable in these settings, trial off by adjusting the ventilator to lung protective ventilation settings (rate, plateau pressure, PEEP, and FiO_2_). Maintain blood flow and anticoagulation, stop the sweep gas, and cap off the oxygenator. If SaO_2_ >95% and PaCO_2_ < 50 mmHg × 60 min, the cannulas can be removed whenever the patient is ready, but ideally after heparin has been turned off for 30–60 min (69).

In VA-ECMO, the first step is a holistic evaluation of the clinical status of the patient. Stable pulmonary status and euvolemia are particularly important (14). ECMO flow is decreased by approximately 1 L/h over a period of 3–4 h, although the slower rates of weaning at 0.5 L every 6–24 h have been reported (70, 73). The patient should be able to maintain mixed venous saturation >65%, and arterial saturation of >90% with an ECMO flow <1.5 L/min (70). In case of decompensation signs, the bridge is clamped, and the patient is placed back on full support (70, 74).

## Conclusions

An increasing number of successful ECMO treatment cases for acute toxic inhalations have been reported. However, the randomized clinical trials are needed to elucidate the survival benefits and to help develop the clinical guidelines and indications for ECMO initiation in acute poisoning. Although the evidence for the clinical applications of ECMO is mainly derived from the retrospective studies, case reports, and case series, we conclude that ECMO is a potential salvage therapy for severe ARDS and refractory cardiogenic shock or cardiac arrest induced by severe toxicological exposures. However, it should be noted that ECMO is a bridge to recovery, to a more durable bridge, to a definitive treatment, or to a better clinical decision, and is a powerful tool that should be used judiciously. Furthermore, all the caregivers involved in the poisoning treatment should be educated on the potentially lifesaving ECMO technology, its indications, complications, and weaning.

## Author Contributions

DY, ZL, and SJ conceptualized and wrote the manuscript. ZXL, PL, LF, ZL, and SJ treated the patient as described in this study. ZL and SJ revised the manuscript. All authors contributed to the article and approved the submitted version.

## Funding

This study was financially supported by the Research Grant for Public Health Key Discipline of Shanghai Municipality, China (No. GWV-10.1-XK26).

## Conflict of Interest

The authors declare that the research was conducted in the absence of any commercial or financial relationships that could be construed as a potential conflict of interest.

## Publisher's Note

All claims expressed in this article are solely those of the authors and do not necessarily represent those of their affiliated organizations, or those of the publisher, the editors and the reviewers. Any product that may be evaluated in this article, or claim that may be made by its manufacturer, is not guaranteed or endorsed by the publisher.
